# Neuroimaging Metrics of Drug and Food Processing in Cocaine-Dependence, as a Function of Psychopathic Traits and Substance Use Severity

**DOI:** 10.3389/fnhum.2018.00350

**Published:** 2018-09-04

**Authors:** William J. Denomme, Isabelle Simard, Matthew S. Shane

**Affiliations:** ^1^The Clinical Affective Neuroscience Laboratory for Discovery and Innovation, Faculty of Social Science and Humanities, University of Ontario Institute of Technology, Oshawa, ON, Canada; ^2^The Mind Research Network, The University of New Mexico, Albuquerque, NM, United States

**Keywords:** psychopathy, neural processing, fMRI, substance dependence, reward, drug and food processing

## Abstract

Previous studies suggest that psychopathic traits commonly present as comorbid with substance use disorders. Moreover, neuroimaging and psychometric findings suggest that psychopathic traits may predispose individuals to a sensitized reward response to drugs. Given that substance use disorders are characterized by a neurocognitive bias toward drug-reward relative to non-drug reward, it is possible that heightened psychopathic characteristics may further predispose to this processing bias. To evaluate this possibility, we assessed psychopathic traits (measured using the PCL-R; Hare, 2003) in 105 probationers/parolees and evaluated the relationship between PCL-R scores, lifetime duration of drug use, and biases in neural response to drug- compared to food-related videos. Psychopathic traits (potentially driven by interpersonal/affective traits) were positively correlated with drug > food reactivity within the right insula and left amygdala. In addition, psychopathic traits modulated the relationship between drug use and drug > food reactivity within the left dorsomedial prefrontal cortex, right insula, and left caudate nucleus. Specifically, lifetime duration of drug use correlated positively with drug > food reactivity in participants with lower levels of psychopathic traits and correlated negatively with drug > food reactivity in individuals with higher levels of psychopathic traits. These results help reconcile prior studies on psychopathy and drug-stimulus processing and provide neurocognitive support for the notion that psychopathic traits serve as an underlying risk factor for substance use disorders. These results suggest that different treatment regimens for substance abuse for individuals with higher or lower levels of psychopathy may be beneficial and suggest that reduction of neurocognitive biases to drug-related stimuli may offer useful targets for future treatment protocols.

## Introduction

Psychopathic individuals have frequently been characterized as impulsive and irresponsible risk takers, with an altered sensitivity to reward and reward-related stimuli (Cleckley, 1941; Mitchell et al., 2002; Hare, 2003; Ross et al., 2007; Wallace et al., 2009; Morgan et al., 2011; Hopley and Brunelle, 2012; Beszterczey et al., 2013; Dean et al., 2013; Salim et al., 2015). Given that these are also characteristics that predict initial and prolonged drug use (Woicik et al., 2009; Koob and Volkow, 2010; Dissabandara et al., 2014; Leeman et al., 2014), it may come as no surprise that psychopathy has been associated with heightened levels of substance use (Kennealy et al., 2007; Coid et al., 2009; Hillege et al., 2010; Cope et al., 2014; Hawes et al., 2015), as well as increased diagnosis of both substance abuse (Hemphill et al., 1994; Mailloux et al., 1997; Cauffman et al., 2009; Sylvers et al., 2011; Jones and Miller, 2012; Colins et al., 2015) and substance dependence (Hart et al., 1991; Hemphill et al., 1994; Walsh et al., 2007; Hopley and Brunelle, 2012).

Beyond this behavioral and diagnostic overlap are additional commonalities. For instance, both disorders appear characterized by dysfunction within common corticolimbic regions underlying reward-related processing (psychopathy: Masui and Nomura, 2011, Blair, 2015; substance use disorders: Koob and Le Moal, 2001). Within adults with psychopathic traits, this dysfunction appears to manifest as consistently heightened sensitivity to a wide variety of rewarding stimuli within the ventral striatum, including monetary (Buckholtz et al., 2010; Bjork et al., 2012; Carré et al., 2013; Pujara et al., 2013) and drug-related (Buckholtz et al., 2010) rewards (though we note that children/adolescent with heightened callous-unemotional traits often show a normal [(Murray et al., 2017; Byrd et al., 2018) or hypersensitive (Veroude et al., 2016) ventral striatal response to reward]. Psychopathic traits have also been associated with increased functional connectivity between the ventral striatum and the dorsomedial prefrontal cortex (DMPFC) in response to monetary rewards (Geurts et al., 2016).

A considerably larger body of work indicates that individuals with substance use disorders also exhibit a heightened reward sensitivity throughout the corticolimbic system (Stewart et al., 2013). However, whereas psychopathic traits appear predictive of broadly increased sensitivity that spans multiple reward categories, individuals with prolonged drug use histories show a sensitivity-profile wherein reward-sensitivity shifts in favor of the individuals’ drug of abuse, particularly within the DMPFC, ACC, striatum, amygdala, and insula (Carter and Tiffany, 1999; Garavan et al., 2000; Kilts et al., 2001; Goldstein et al., 2007; Lubman et al., 2008; Chase et al., 2011; Kühn and Gallinat, 2011; Engelmann et al., 2012; Yalachkov et al., 2012; Ray et al., 2015; Tomasi et al., 2015), at the expense of non-drug rewards [e.g., sex-related (Garavan et al., 2000), monetary (Goldstein et al., 2009)]. This substance-induced decalibration of the reward system has been theorized as central to the development and maintenance of craving, drug-seeking, and compulsive drug use, wherein the individual is motivated to seek out the strong reward properties of the drug, and has difficulty obtaining that level of reward through non-drug rewards (Goldstein and Volkow, 2002, 2011; see also Koob and Le Moal, 1997, 2008a,b; Everitt and Robbins, 2005; Koob and Volkow, 2010).

How these reward-dysfunctions are related and whether they explain the comorbidity between the two disorders remains poorly understood. It is possible that heightened psychopathic traits predisposes to a sensitized reward-response to drugs, which would be related to a reward-processing bias toward drug-related rewards compared to non-drug rewards. While little work has yet been directed toward such issues, one recent study provides preliminary support. In this study, drug-naïve individuals had their neural reactivity evaluated during a controlled amphetamine administration (Buckholtz et al., 2010). Results indicated that impulsive/antisocial psychopathic traits were associated with an increasingly sensitized ventral striatal dopaminergic response to the amphetamine administration. Such a heightened corticolimbic dopamine response to drugs is believed to serve as a catalyst for the development of longer-term neuroplastic changes to drug-related incentive salience, and a resultant processing bias for drug-compared to non-drug-rewards (Robinson and Berridge, 1993, 2000; Koob and Le Moal, 1997; Di Chiara, 1999; Volkow et al., 1999; Goldstein and Volkow, 2002; Wise, 2004; Koob and Volkow, 2010; Pickens et al., 2011; O’Daly et al., 2014; Volkow and Morales, 2015). Thus, the inclination for individuals with heightened psychopathic traits to select highly risky rewards (Mitchell et al., 2002), combined with their initially heightened reward sensitivity (e.g., Bjork et al., 2012), may increase the likelihood of corticolimbic sensitization to drug-related rewards (followed by a substance induced desensitization to non-drug rewards). As a result, they would continue to abuse these drugs and may be more likely to develop substance dependence disorders.

Recently, a small amount of work has begun to investigate this hypothesis by assessing drug-stimulus processing in substance users with varying levels of psychopathy. Cope et al. (2014) assessed the relationship between psychopathy and the neural response to drug-related and neutral stimuli among 137 male offenders meeting the *DSM-IV-TR* (American Psychiatric Association, 2000) criteria for lifetime dependence to heroin, cocaine, or methamphetamines. Results identified a negative correlation between psychopathic traits and neural response to drug versus neutral images in the ACC, putamen, caudate, amygdala, and ventral striatum. Vincent et al. (2017) largely replicated these results utilizing the same stimulus-presentation task in 54 male adolescent offenders (44 of whom had a stimulant use disorder) who manifested a negative correlation between psychopathic traits and neural response to drug versus neutral images in the ACC, amygdala, caudate, hippocampus, insula, and striatum.

While these results seemingly counter our hypothesis, several features of Cope et al. (2014) and Vincent et al. (2017) suggest that additional investigation may be in order. First, both studies used on a non-reward control condition. While this provides a true non-reward baseline, it precludes the ability to determine whether the psychopathy-related reduction in cue-elicited reactivity was specific to drug-related stimuli or could instead be due to a more general reduction in reactivity to all reward-related stimuli (see Versace et al., 2017 for commentary on the pitfalls of neutral conditions). This distinction may be particularly important given that substance use disorders are known to preferentially bias neural systems toward drug-related stimuli and away from other categories of non-drug rewards (i.e., food; Baler and Volkow, 2006; Volkow et al., 2010, 2016; Rubinstein et al., 2011; Schwienteck and Banks, 2015; Schwienteck et al., 2015; Versace et al., 2017). To this end, the present study made use of a carefully matched non-drug reward (i.e., food) condition as our control condition. By including a food-reward condition, the paradigm afforded careful isolation of drug reward-related neural activity from natural reward-related activity. Thus, we could assess whether psychopathic traits are related to an abnormal neural sensitivity to all types of reward, or whether this abnormality is specific to drug-related rewards.

A second potential limitation of Cope et al. (2014) and Vincent et al. (2017) is that they did not include a non-dependent control condition. While we would not necessarily expect psychopathy to mediate neural responses to drug cues in a non-dependent group, the inclusion of this group can confirm the specificity of any mediated response in individuals with a previous drug-use history. Such specificity may provide additional clues toward the etiological basis of any observed psychopathy-related influences. To this end, we recruited both dependent and non-dependent subjects into the present study.

We hypothesized drug- and food-related hemodynamic signal-change differences in the insula, DMPFC, ACC, amygdala, and the striatum between dependent and non-dependent groups. We additionally hypothesized that psychopathic traits would mediate neural reactivity to drug versus food stimuli in the dependent group. Finally, we predicted that psychopathic traits would interact with substance use, such that the influence of psychopathic traits on drug and food processing would be modulated by the level of substance use.

## Materials and Methods

### Participants

Our sample consisted of 105 adult probationers/parolees (70 males) residing in the great Albuquerque, New Mexico area. Participants were recruited through probation/parole offices, halfway houses, and drug treatment centers, as well as through targeted advertisements in local print and online classifieds. Classified ads specifically targeted probationers/parolees who did, and did not, meet *DSM-IV-TR* diagnostic criteria for lifetime cocaine dependence. Exclusion criteria included loss of consciousness for longer than 30 min, lifetime history of psychotic disorder, diagnosis of major depressive disorder within last 6 months, and standard MR-related exclusion criteria including metallic implants, permanent retainer or braces, irremovable piercings, other metal irremovable metallic objects, and pregnancy. Diagnosis of anxiety disorders, including obsessive-compulsive disorder, were documented but not used as exclusion criterion. This study was approved by the Institutional Review Board of the University of New Mexico and the Research Ethics Board of the University of Ontario Institute of Technology and carried out in accordance with their recommendations. All subjects gave written informed consent in accordance with the Declaration of Helsinki.

### Clinical/Forensic Measures

#### Cocaine Dependence

Lifetime history of cocaine dependence was diagnosed via the Structured Clinical Interview for *DSM-IV-TR* Axis I Disorders (SCID-I/P; First et al., 2002). Psychiatric symptoms of all disorders are coded 1 to 3, representing absent (1), subthreshold (2), or threshold/present (3). As per SCID I/P procedures, a diagnosis of cocaine dependence required that the participants score “3” on at least three of seven diagnostic criteria. Highly trained graduate research personnel conducted each interview, under the guidance of a senior SCID trainer (R.C.; see “Acknowledgments”).

#### Psychopathic Traits

The Psychopathy Checklist-Revised (PCL-R; Hare, 2003) was utilized to measure psychopathic traits. The PCL-R is widely considered the gold-standard instrument to diagnose psychopathy (Lynam and Gudonis, 2005), and has demonstrated good reliability and construct validity in substance abuse patients (Alterman et al., 1993; Rutherford et al., 1996, 1997) and offenders (Cooke and Michie, 1997; Shine and Hobson, 1997; Sullivan et al., 2006; Poythress et al., 2010; Neves et al., 2011). For the present study, PCL-R scores were calculated based on an in-depth interview administered by highly trained research personnel (trained by MS); no subsequent file review was undertaken. It consisted of 20 items scored 0–2, with scores ranging from 0 to 40. Both Total and Factor scores were calculated and evaluated with regard to primary variables of interest. Factor 1 contained eight items assessing interpersonal and affective deficits; Factor 2 contained 10 items assessing lifestyle and antisocial deficits.

#### Drug Use

In addition to SCID-I/P diagnoses of substance dependence disorders, a trained examiner also administered a modified version of the Addiction Severity Index-Expanded (ASI-X; McLellan et al., 1992) to assess the frequency and duration of participants’ regular substance use history. Following data collection, three composite drug use scores were calculated by summing the total number of years of use of drugs that fell into one of three categories: Major Drugs (e.g., cocaine, heroin, methamphetamines), Minor Drugs (e.g., cannabis, nicotine, hallucinogens), and Alcohol (see Claus and Shane, 2018). For example, if a participant used cocaine for 5 years, methamphetamines for 5 years, and heroin for 3 years, the effective rate of Major Drug use was calculated as 13 years.

### Cue-Elicited Craving Task

Participants performed two identical runs of a cue-elicited block-design craving task randomly sequenced and presented via E-Prime 2.0 (Psychology Software Tools Inc., 2012: Psychology Software Tools, Pittsburgh, PA, United States). Participants were presented with 29 videos ranging from ∼10,000–14,000ms in duration. Videos were organized into two categories: 15 videos depicting people preparing or using cocaine/crack (DRUG); or 14 videos depicting people preparing/eating various foods (FOOD). The distinction between use and preparation of drugs and food was made for purposes outside the scope of this study – thus they were collapsed for all analyses within the current study. Participants were simply asked to watch the videos and were not required to make any formal assessments during video playback. However, following each video, participants were prompted to rate their level of craving on a scale from 1 (lowest) to 4 (highest) on a four-button keypad. Following a jittered inter-trial interval (2500, 3500, and 5000 ms) to aid deconvolution from the standard hemodynamic response function (HRF), the next video was presented.

### Image Acquisition Parameters and Preprocessing

Participants were scanned using a Siemens 3T TrioTim MRI scanner with advanced SQ gradients (max slew rate 200 T/m/s) at The Mind Research Network imaging center. Whole-brain T2^∗^-weighted images were acquired from a 16-element phased-array head coil and an iPAT echo-planar imaging (EPI) gradient-echo pulse sequence (TR = 2000 ms; TE = 29 ms). Image acquisition utilized a 75° flip angle and created a 24 cm × 24 cm FOV on a 64 × 64 matrix, generating 33 slices of 3.5 mm covering the entire brain (roughly 150 mm) and creating a 3.4 mm × 3.4 mm in-plane resolution. Head motion was limited using padding and restraints.

Brain images were preprocessed using a custom pipeline with Statistical Parametric Mapping 5 (SPM5^1^. Motion parameters were collected along six dimensions (x, y, z; pitch, yaw, roll) and corrected using INRIAlign (Freire et al., 2002), which applies an algorithm with a non-quadratic function, unbiased by local signal changes, that reduces the influence of intensity differences between slice images. No participants demonstrated head movement exceeding 5 mm. Images were then normalized according to the standard single-subject MNI template and smoothed with a 10 mm Full Width Half-Maximum (FWHM) Gaussian smoothing kernel.

### Data Analytic Strategies

Psychometric data and correlations with psychometric data were analyzed within the Statistical Package for the Social Sciences 24 (SPSS 24; Industrial Business Machines [IBM], 2016).

First-level neuroimaging analyses were performed using a custom SPM5 analysis script to extract blood-oxygen-level-dependent (BOLD) signals throughout the task. The first-level design matrix included video presentation as one event separated into four conditions (depicting drug preparation, drug use, food preparation, and food use). Mean functional images of blood oxygen-level-dependent signals throughout the whole brain were extracted from each of the four conditions. This model also included six movement parameters (x, y, z, yaw, pitch, roll) that were covaried out of the model as variables of no interest. T-contrasts were then computed at the first level to assess changes in hemodynamic response during the duration of the DRUG and FOOD videos relative to baseline and to each other.

Second-level neuroimaging analyses were conducted using a mixed-model flexible-factorial ANOVA in SPM12. Subject and Video Type (DRUG, FOOD) were included as within-group factors, and Group (Dependent, Non-Dependent) was included as a between-group factor. Higher-order main effects of VideoType and Group, the Group^∗^VideoType interaction, and targeted T-contrasts to evaluate between- and within-group differences in neural responses to DRUG > FOOD were interrogated within the flex-factorial model. All second level analyses were conducted with and without age as a null covariate; results reported below were modeled without age.

Of particular interest was the extent to which PCL-R scores and/or substance use severity would predict the magnitude of any DRUG > FOOD processing bias identified within the Dependent group. To investigate this, multiple linear regression models were undertaken, with PCL-R Total Scores, Major Drug Use, and the PCL-R^∗^Major Drug Use interaction term, included as regressors to predict BOLD response in the DRUG > FOOD contrast. These regressions were run separately among Dependent and Non-dependent groups, however, results focus on the Dependent results, as these were of primary theoretical importance. Similar regression models were also conducted with Factor 1 and Factor 2 scores as regressors to evaluate the unique influence of interpersonal/affective and lifestyle/antisocial traits.

Whole-brain results were interpreted using an uncorrected threshold of *p* < 0.001, combined with an extended cluster threshold of 132 voxels (equivalent to a *p* < 0.05 [FWE] threshold) based on a series of Monte-Carlo simulations run through the Alpha Simulator (AlphaSim) in the Resting-State fMRI Data Analysis Toolkit (REST; Song et al., 2011).

### ROI Analysis

In addition to whole-brain analyses, small-volume correction (*p* < 0.05 FWE-svc) was used to assess activity within six regions of interest (ROIs) : right insula (*x* = 40, *y* = -8, *z* = -18), left ACC (*x* = -6, *y* = 4, *z* = 44), left DMPFC (*x* = -5, *y* = 46, *z* = 34), right ventral striatum (*x* = 11, *y* = 13, *z* = -7), left amygdala (*x* = -32, *y* = 0, *z* = -27), and left caudate nucleus (*x* = -9, *y* = -4, *z* = 12). All central coordinates were obtained from a recent meta-analysis, which identified these regions within individuals with cocaine use disorders as regions that show specifically reactivity following presentation of cocaine-related cues (Kühn and Gallinat, 2011). A 6 mm spherical search space was used for subcortical ROIs (i.e., ventral striatum, amygdala, and caudate) while a 10 mm sphere was used for cortical ROIs (i.e., insula, ACC, DMPFC).

Parameter estimates of signal change to DRUG and FOOD videos extracted from each ROI were evaluated via ANOVA and correlational models in SPSS. Parameter estimates from peak-voxel coordinates, and also average parameter estimates from all coordinates within the ROI, were evaluated, exhibiting identical similar results. Within the manuscript, we report peak-voxel coordinate analyses and results.

## Results

### Descriptive Statistics and Correlations Between Variables of Interest

Descriptive statistics of all clinical/forensic variables are displayed in **Table 1**; correlations between these variables are displayed **Table 2**. The mean sample age was 35.86 (*SD* = 9.04; range = 21–59), and the mean IQ was 105.47 (*SD* = 12.13; range = 77–140). As may be expected, Dependent participants reported greater lifetime drug use than Non-dependent participants, *t* = 6.70, *p* < 0.001 particularly with regard to major, *t* = 8.08, *p* < 0.001, but not minor, *t* = 0.22, *p* = 0.82, drug use. Moreover, Dependent participants had higher PCL-R Total, *t* = 4.57, *p* < 0.001, and Factor (Factor 1, *t* = 3.42, *p* = 0.001; Factor 2, *t* = 4.14, *p* < 0.001) scores than Non-dependent participants. Finally, Dependent participants also had a higher mean age, *t* = 2.53, *p* = 0.013.

**Table 1:** Descriptive statistics and group-level differences in clinical/forensic variables.

Variable	Whole sample	Dependent group	Non-dependent group	*t*
Age	35.86 (9.04)	38.28 (8.56)	33.90 (9.02)	2.53ˆ*
IQ	105.47 (12.13)	105.47 (12.13)	105.91 (11.69)	0.418
Major drug use	7.44 (8.26)	13.36 (8.40)	2.64 (3.87)	8.08ˆ***
Minor drug use	21.67 (16.26)	22.06 (16.38)	21.35 (16.29)	0.224
Alcohol use	8.32 (9.86)	11.51 (10.50)	5.74 (8.57)	3.10ˆ**
PCL-R Total	18.78 (7.33)	22.11 (6.83)	16.08 (6.62)	4.57ˆ***
Factor 1	6.40 (3.39)	7.60 (3.20)	5.43 (3.26)	3.42ˆ**
Factor 2	11.08 (4.20)	12.83 (3.91)	9.66 (3.91)	4.14ˆ***

*t-Values represent magnitude of difference between Dependent and Non-dependent participants. Unbracketed values represent means, while bracketed values represent standard deviations. ^∗^*p* < 0.05, ^∗∗^*p* < 0.01, ^∗∗∗^*p* < 0.001.*

**Table 2:** Correlations between clinical/forensic variables among dependent and non-dependent participants.

	1	2	3	4	5	6	7	8
**Dependent group**
(1) Age	–	–0.31ˆ*	0.41ˆ**	–0.27	0.16	0.18	0.24	0.13
(2) IQ		–	–0.17	0.09	–0.12	–0.10	0.02	–0.21
(3) Major drug use			–	–0.10	0.12	0.33ˆ*	0.17	0.46ˆ**
(4) Minor drug use				–	–0.06	0.04	0.13	0.02
(5) Alcohol use					–	0.10	0.06	0.12
(6) PCL-R Total						–	0.88ˆ***	0.89ˆ***
(7) Factor 1							–	0.62ˆ***
(8) Factor 2								–
**Non-dependent group**
(1) Age	–	–0.10	0.13	–0.07	0.38ˆ**	–0.15	–0.06	–0.15
(2) IQ		–	–0.14	0.04	–0.30ˆ*	–0.10	0.05	–0.20
(3) Major drug use			–	–0.11	–0.16	0.11	–0.02	0.16
(4) Minor drug use				–	–0.09	–0.09	–0.04	–0.13
(5) Alcohol use					–	0.21	0.22	0.12
(6) PCL-R total						–	0.85ˆ***	0.87ˆ***
(7) Factor 1							–	0.53ˆ***
(8) Factor 2								–

*^∗^*p* < 0.05, ^∗∗^*p* < 0.01, ^∗∗∗^*p* < 0.001.*

### Baseline Sensitivity to DRUG and FOOD Stimuli

Neural responses to DRUG and FOOD stimuli were first evaluated using a 2 (VideoType) × 2 (Group) flexible-factorial ANOVA. This analysis revealed significant main effects of both Group and VideoType that spanned across frontal, temporal, parietal, occipital, and limbic cortices (see **Table 3**). These main effects were influenced by a significant Group × VideoType effect, which presented within several clusters that encompassed the left ACC, right insula, right ventral striatum, left amygdala, and right hippocampus.

**Table 3:** Higher-order ANOVA results.

Region	Hemi.	MNI (*x, y, z*)	*F*	Cluster size
**Main effect of Group**				
Angular gyrus	R	60, –54, 12	29.12^∗^	140
	R	57, –60, 18	20.81^∗^	
Middle temporal cortex	R	45, –42, –6	16.32	
Middle occipital cortex	R	48, –72, 27	23.23^∗^	192
	R	45, –78, 18	22.52^∗^	
Calcarine cortex	R	6, –69, 12	22.71^∗^	
Ventral striatum	R	12, 12, 0	6.89†	27
Amygdala	L	–9, –6, 12	7.14†	22
**Main effect of Video Type**				
Middle occipital cortex	L	–18, –99, 3	58.24^∗^	193
Fusiform gyrus	L	–30, –78, –18	33.95^∗^	
Middle occipital cortex	R	12, –99, 9	56.18^∗^	268
Fusiform gyrus	R	30, –81, –15	17.57	
Superior occipital cortex	L	–24, –78, 30	39.36^∗^	244
Superior parietal cortex	R	24, –54, 60	38.22^∗^	218
Middle frontal cortex	R	45, 18, 3	28.37^∗^	768
Inferior temporal cortex	L	–45, –45, –9	19.07	180
ACC	L	–9, 6, 54	15.91†	70
DMPFC	L	–3, 36, 36	9.50†	35
Ventral striatum	R	18, 12, –6	6.94†	1
**Interaction effect**				
Orbitofrontal cortex	R	42, 36, –3	21.75^∗^	342
Insula	R	48, 15, –12	15.12	
	R	33, 21, –12	14.01	
Middle frontal cortex	R	51, 15, 18	20.11^∗^	230
Superior frontal cortex	R	36, 15, 36	15.10	
DMPFC	R	51, 33, 18	14.71	
ACC	L	–6, 18, 21	19.47^∗^	349
	L	–6, 27, 6	18.61	
Middle temporal cortex	L	–63, –24, –12	19.27^∗^	159
	L	–57, –6, –6	17.83	
Superior temporal cortex	L	–48, 12, –18	12.21	
Insula	R	36, 0, –21	9.48†	73
Ventral striatum	R	6, 12, –6	8.48†	15
	R	12, 18, –6	6.90†	
Amygdala	L	–36, 0, –24	7.87†	27

*All regions show significant activity at *p*(uncorr) < 0.001; ^∗^*p*(FWE) < 0.05; † *p*(svc-FWE) < 0.05; ACC = Anterior cingulate cortex; DMPFC = Dorsomedial prefrontal cortex.*

**FIGURE 1:**
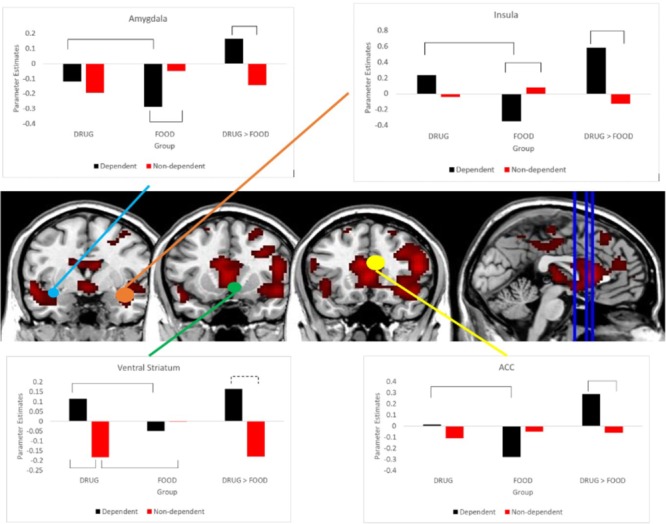
Differential sensitivity to DRUG and FOOD videos between groups. Bar-charts demonstrate differences in DRUG and FOOD reactivity between the Dependent and Non-dependent groups. Brackets indicate significant differences at *p* < 0.05. MRIcron images display intensity thresholds ranging from T = 6.17 – 22.56. Coronal slices register to MNI coordinate y = 0, y = 12, and y = 18, respectively.

To evaluate the nature of this interaction effect, parameter estimates from these five ROIs were extracted from DRUG and FOOD trials and entered into mixed-factor ANOVA models in SPSS. As seen in **Figure 1**, Bonferroni-controlled *t*-tests indicated that the Dependent group exhibited greater DRUG-related activity within the right ventral striatum ROI, *t* = 2.42, *p*(FWE) = 0.017 and left ACC, *t* = 1.91, *p*(FWE) = 0.058; and reduced FOOD-related activity within the left ACC, *t* = 2.48, *p*(FWE) = 0.015, left amygdala, *t* = 2.13, *p*(FWE) = 0.036, and right insula, *t* = 1.94, *p*(FWE) = 0.055.

#### Relative Sensitivity to DRUG Versus FOOD Stimuli

To assess participants’ *relative* responses to DRUG versus FOOD stimuli, we conducted separate within-sample *t*-tests on the DRUG > FOOD contrast in each of the Dependent and Non-dependent groups (see **Table 4**). As hypothesized, Dependent participants exhibited significantly greater DRUG than FOOD reactivity within the right insula, left ACC, right ventral striatum, and left amygdala ROIs, as well as the left DMPFC. Greater FOOD > DRUG reactivity was only observed within the bilateral occipital cortex. In contrast, Non-dependent participants did not exhibit any regions with greater DRUG than FOOD reactivity, yet demonstrated greater reactivity to FOOD than DRUG stimuli within the right insula, right ventral striatum, and left caudate nucleus (see **Supplementary Table S1**). Between-group differences were evaluated via a between-group *t*-test, which confirmed that the Dependent group exhibited significantly greater DRUG > FOOD bias than the Non-dependent group within the right insula, left DMPFC, right ventral striatum, left amygdala, and left DMPFC.

**Table 4:** Within- and between-group neural activity to Drug and Food videos.

Region	Hemi.	MNI (*x, y, z*)	*t*	Cluster size
**Drug > Food in the Dependent Group**				
ACC	L	–9, 21, 18	6.07^∗^	2980
Lateral prefrontal cortex	R	45, 21, 3	5.74^∗^	
Lateral prefrontal cortex	L	–42, 21, –3	5.07^∗^	615
Dorsolateral prefrontal cortex	L	–45, 39, 15	4.95^∗^	
Middle frontal cortex	L	–57, 21, 15	4.73^∗^	
Middle occipital cortex	R	9, –99, 12	5.05^∗^	226
Fusiform gyrus	R	30, –81, –15	4.08	
Angular gyrus	R	48, –48, 42	4.84^∗^	150
Postcentral gyrus	L	–12, –21, 57	4.27^∗^	147
Precentral gyrus	L	–6, –9, 54	3.61	
Insula	R	30, –6, –15	2.82†	44
	R	36, 0, –15	2.69†	
ACC	L	–9, 9, 51	4.00†	117
	L	–9, –6, 48	2.86†	
DMPFC	L	0, 39, 33	3.46†	96
	L	–3, 36, 36	3.44†	
Ventral striatum	R	18, 12, –6	2.87†	14
Amygdala	L	–33, –3, –24	2.35†	23
**Drug > Food in the Non-dependent Group**				
No significant results				
**Drug > Food in the Dependent > Non-dependent**				
Ventrolateral prefrontal cortex	R	42, 36, –3	4.66^∗^	1455
Middle frontal cortex	R	51, 15, 18	4.48^∗^	
ACC	L	–6, 18, 21	4.41^∗^	
Middle temporal cortex	L	–63, –24, –12	4.39^∗^	249
	L	–57, –6, –6	4.22^∗^	
Superior temporal cortex	L	–48, 12, –18	3.49	
Orbitofrontal cortex	L	–42, 24, –6	3.87	177
Lateral prefrontal cortex	L	–42, 36, 12	3.86	
Ventrolateral prefrontal cortex	L	–36, 39, 0	3.32	
Insula	R	36, 0, –21	3.08**†**	117
DMPFC	L	–3, 45, 24	2.71**†**	89
Ventral striatum	R	6, 12, –6	2.91**†**	22
	R	12, 18, –6	2.63**†**	
Amygdala	L	–39, 0, –21	2.81**†**	32

*All regions show significant activity at *p*(uncorr) < 0.001; ^∗^*p*(FWE) < 0.05; **†***p*(svc-FWE) < 0.05; DMPFC = Dorsomedial prefrontal cortex; ACC = Anterior cingulate cortex.*

### Influence of PCL-R Scores and Drug Use Severity

Given that between-group analyses confirmed that cocaine-dependent individuals were characterized by a DRUG > FOOD processing bias compared to Non-dependent participants, we next evaluated the extent to which psychopathic traits and substance use history would relate to this processing bias. To this end, we undertook a series of regression models in SPM12, entering PCL-R Total scores, years of Major Drug Use, and the PCL-R × Major Drug Use interaction term, as regressors predicting DRUG > FOOD reactivity. We ran models within both Dependent and Non-dependent groups but focused primarily on the Dependent group (**Table 5**) given the unknown response to drug cues within the Non-dependent group (see **Supplementary Table S2** for results in the Non-dependent group).

**Table 5:** Multiple regression results: total PCL-R scores, Major Drug Use, as predictors of DRUG > FOOD-related hemodynamic activity among the dependent group.

Region	Hemi.	MNI (*x, y, z*)	*t*	Cluster size
**PCL-R**				
*Positive*				
Insula	R	36, –15, –15	3.46†	151
	R	36, –12, –21	3.34†	
Amygdala	L	–30, –3, –24	2.95†	23
*Negative*				
No significant results				
**Major Drug Use**				
No significant results				
**PCL-R ^∗^ Major Drug Use**				
DMPFC	R	24, 30, 33	5.28^∗^	150
Superior frontal cortex	R	15, 27, 48	3.61	
Insula	R	39, –9, –9	2.78†	63
DMPFC	L	–15, 42, 36	3.40†	168
	L	3, 48, 30	2.72†	
Caudate	L	–3, –3, 12	3.72†	33
	L	–6, 0, 15	3.63†	

*All regions show significant activity at *p*(uncorr) < 0.001; ^∗^*p*(FWE) < 0.05; **†***p*(svc-FWE) < 0.05; DMPFC = Dorsomedial prefrontal cortex.*

Results indicated that PCL-R scores, but not Major Drug Use, were positively correlated with activity within several regions, including right insula and left amygdala ROIs. We followed up these regressions by correlating PCL-R scores with parameter estimates from these ROIs and observed positive correlations between PCL-R scores and DRUG-related activity in the right insula, *r* = 0.31, *p* = 0.037, and negative correlations with FOOD-related activity in the right insula, *r* = -0.34, *p* = 0.021, and left amygdala, *r* = -0.33, *p* = 0.025.

**FIGURE 2:**
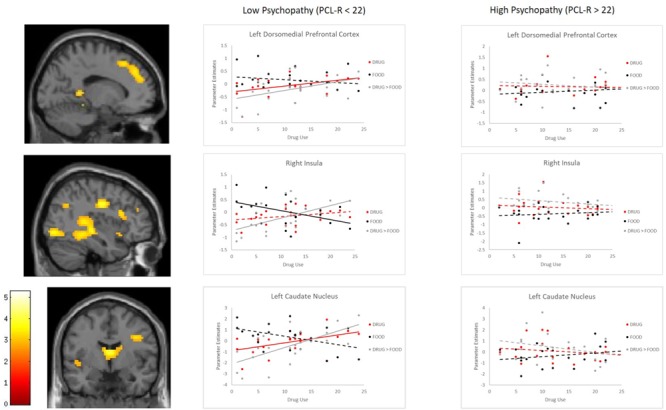
Correlations between parameter estimates and Major Drug Use among Dependent participants with high and low PCL-R scores. Scatterplots demonstrate correlations between Major Drug Use and parameter estimates of neural activity in response to DRUG and FOOD videos, as well as their calculated difference in activity (DRUG > FOOD). Solid lines indicate significant correlations at *p* < 0.05. Dotted lines indicate non-significant relationships. Brain images are displayed at a significance threshold of *p*(uncorr) < 0.005, with an extended cluster threshold of *k* = 30.

In addition, we observed a significant PCL-R × Major Drug Use interaction within the left DMPFC, bilateral insula, and left caudate nucleus. In order to decipher these interaction effects, we separated our Dependent group into high and low PCL-R groups (via median split; median PCL-R = 22). The high PCL-R group exhibited greater DRUG > FOOD activity within left DMPFC, *t* = 2.62, *p* = 0.012, and right insula, *t* = 3.23, *p* = 0.002. We then evaluated correlations between Major Drug Use composite scores and parameter estimates within each group separately. Parameter estimates from FOOD trials were subtracted from DRUG trials to obtain DRUG > FOOD reactivity estimates. Within the low PCL-R group, Major Drug Use correlated positively with DRUG > FOOD reactivity within the left DMPFC, *r* = 0.59, *p* = 0.003, right insula, *r* = 0.56, *p* = 0.005, and left caudate, *r* = 0.61, *p* = 0.002. In contrast, the high PCL-R group exhibited a marginally significant negative correlation between Major Drug Use and DRUG > FOOD reactivity within the right insula *r* = -0.38, *p* = 0.071 and left caudate nucleus, *r* = -0.37, *p* = 0.077. These correlations were followed by correlations between Major Drug Use and parameter estimates extracted from FOOD and DRUG trials relative to baseline. Among the low PCL-R group, Major Drug Use correlated positively with DRUG-related left DMPFC, *r* = 0.53, *p* = 0.009, and left caudate, *r* = 0.44, *p* = 0.036, activity and correlated negatively with FOOD-related right insula, *r* = -0.46, *p* = 0.028, and left caudate. *r* = -0.46, *p* = 0.026, activity. The high PCL-R group did not exhibit any significant correlations. See **Figure 2** for a visual depiction.

### Influence of Psychopathy Factors and Drug Use Severity

Finally, to better understand how PCL-R factors differentially influenced neural reactivity, we undertook additional regression models with PCL-R Factor scores (and Major Drug Use) entered as separate regressors to predict activity in the DRUG > FOOD contrast. Within the Dependent group, these analyses indicated that Factor 1 was associated with activity in several regions, including the right insula, right ventral striatum, and left amygdala (**Table 6**; see **Supplementary Table S2** for results in the Non-dependent group). Analysis of parameter estimates from each of the FOOD and DRUG contrasts confirmed that Factor 1 scores were positively correlated with DRUG-related activity within right insula, *r* = 0.39, *p* = 0.006, and left amygdala, *r* = 0.30, *p* = 0.042, and negatively correlated with FOOD-related activity within ventral striatum, *r* = -0.29, *p* = 0.049, and left amygdala, *marginal r* = -0.26, *p* = 0.084. Factor 2 showed no associated with DRUG > FOOD reactivity, and no interaction effects between Major Drug Use and Factor scores were identified.

**Table 6:** Multiple regression results: PCL-R factor scores, major drug use, as predictors of DRUG > FOOD-related hemodynamic activity among the dependent group.

Region	Hemi.	MNI (*x, y, z*)	*t*	Cluster size
**Factor 1**				
*Positive*				
Cerebellum	R	39, –63, –48	4.71	116
	R	21, –78, –42	3.93	
Parahippocampal gyrus	L	–24, –21, –27	4.71	285
Fusiform gyrus	L	–27, –36, –21	4.69	
	L	–39, –60, –12	3.57	
Fusiform gyrus	R	27, –39, –18	4.46	200
	R	36, –33, –18	4.18	
Lingual gyrus	R	33, –51, –3	3.52	
Insula	R	39, –12, –24	4.06†	164
Ventral striatum	R	9, 9, –9	3.12†	32
	R	9, 15, –6	3.10†	
Amygdala	L	–30, –3, –24	2.89†	26
*Negative* No significant results				
**Factor 2**				
No significant results				
**Use**				
No significant results				
**Factor 1^∗^Major Drug Use**				
No significant results				
**Factor 2^∗^Major Drug Use**				
No significant results				

*All regions show significant activity at *p*(uncorr) < 0.001; ^∗^*p*(FWE) < 0.05; **†***p*(svc-FWE) < 0.05.*

## Discussion

We used an fMRI cue-elicited craving task to assess neural reactivity to drug-related and food-related stimuli within individuals with and without a cocaine dependence disorder. We first noted a neural processing bias for drug-related relative to food-related stimuli among cocaine-dependent participants relative to Non-dependent participants, within a variety of regions including the ACC, DMPFC, amygdala, ventral striatum, and insula. These results are consistent with a large body of neuroimaging work which has demonstrated increased corticolimbic responsivity to drug-related rewards compared to either neutral (Garavan et al., 2000; Kilts et al., 2001; Bonson et al., 2002; David et al., 2005; Chase et al., 2011; Kühn and Gallinat, 2011; Engelmann et al., 2012; Ray et al., 2015), or non-drug rewards (Garavan et al., 2000; George et al., 2001; Goldstein et al., 2009). Specifically, analysis of parameter estimates indicated that certain corticolimbic regions (i.e., right ventral striatum) exhibited increase neural sensitivity to cocaine-related stimuli, and other corticolimbic regions (i.e., left ACC and left amygdala) exhibited decreased sensitivity to food-related stimuli. Together, these findings offer further support for the notion that individuals with substance dependence disorders exhibit a specifically heightened reward response for drug-related rewards, and a concomitant decrease in reactivity to non-drug rewards (see Goldstein and Volkow, 2002, 2011). Contemporary models of addiction (i.e., I-RISA: Goldstein and Volkow, 2002; antireward-theory: Koob and Le Moal, 1997; Blum et al., 2000; Franken, 2003) argue that destabilization of this neural sensitivity may contribute to substance-dependent individuals’ engagement in habitual, uncontrollable drug-seeking behavior.

Of particular interest was the extent to which either psychopathic traits or duration of substance use history would influence the magnitude of this neural processing bias. No main effect of substance use history was identified, suggesting that the addiction-related processing bias for drug-related stimuli may develop early in the addiction cycle (see Koob and Le Moal, 1997), and remain stable with prolonged use. In contrast, a main effect of psychopathic traits was identified within the right insula and left amygdala, such that an increase in psychopathic traits was associated with a more severe Drug > Food bias within these regions. Analysis of parameter estimates indicated that within the right insula, psychopathic traits were associated with an increase in drug-related responsivity and a decrease in food-related responsivity; in the left amygdala, a decrease in food-related responsivity were found. These findings support the hypothesis that psychopathic traits would moderate the magnitude of drug-related reward sensitivity in substance abusing individuals. Considering that the insula has been noted to be involved in the interoceptive reward-processing of drug use (Weiss, 2005; Naqvi and Bechara, 2009, 2010; Koob and Volkow, 2016), and the amygdala is involved in salience attribution to rewarding stimuli (Weiss, 2005; Ding et al., 2013; Lee et al., 2013; Murray et al., 2017), psychopathic traits may impart an enhanced incentive sensitization to drugs and drug-related interoceptive reward, as well as an enhanced incentive desensitization to food-related reward.

These results run somewhat counter to the results of Cope et al. (2014) and Vincent et al. (2017), which reported decreased drug-related reactivity with increasing levels of psychopathy. One difference worth noting is that our study focused only on cocaine dependence and utilized a cocaine-cue craving task, whereas Cope et al. (2014) utilized methamphetamine, heroin, and cocaine users, and Vincent et al. (2017) focused on stimulant users. However, we believe that a more important distinction between our study and these prior reports is that our study made use of a non-drug reward (food) control condition (rather than a neutral control condition). By using such a non-drug reward control condition, the present study was able to interrogate the extent to which psychopathy-induced variation in neural reactivity to drug-related stimuli was due to consistent changes in reactivity to all forms of rewarding stimuli or was instead specific to the processing of drug-related stimuli (see Versace et al., 2017 for discussion of problems with neutral control conditions). The present results appear to support the latter hypothesis: individuals with heightened psychopathic traits showed greater Drug > Food processing biases, suggestive of particularly strong desensitization of non-drug rewards. It is likely that the use of a neutral control condition in previous studies would have had difficulty identifying this distinction, and that a negative Drug > Neutral bias may preclude a positive Drug > Non-drug bias. For instance, psychopathic traits could associate with a decrease in drug-related reward-processing, while also associating with a greater decrease in non-drug reward-sensitivity. Future research should evaluate whether the present findings, and those of Cope et al. (2014) and Vincent et al. (2017) can be reconciled along such lines.

Interestingly, we also observed an interaction between psychopathic traits and substance use history, such that the positive correlations between substance use history and Drug > Food processing occurred only within participants with a low level of psychopathic traits. In contrast, we observed a marginally significant negative correlation within participants with a high level of psychopathic traits. These results suggest that the development of a specific affinity toward drug-related rewards in substance users may only be apparent when in combination with a low level of psychopathic traits. In highly psychopathic individuals, on the other hand, we observed a decreased sensitivity to drug-related rewards. While highly psychopathic individuals, characterized by a high sensitivity to rewarding stimuli (Bjork et al., 2012), initially exhibit this drug-specific reward sensitivity, they may begin to exhibit a premature desensitization of this reward-processing bias with increasing substance use. This raises further question about the implication of psychopathic traits on the development and maintenance of substance use disorders.

Further study should be allocated toward the nature of the comorbidity between psychopathy and addiction. The fact that the highly psychopathic group, as in the previous literature (Cope et al., 2014), observed decreases in reward-related reactivity rather than increases, raises questions about how reward dysfunction in psychopathic individuals moderates the development and maintenance of substance dependence. Two possibilities may explain why those with heightened psychopathic traits would be associated with decreases in drug-cue reactivity yet increases in substance use disorders. One is that with increasing substance use, highly psychopathic individuals may begin to lose interest in the drug, possibly due to a decrease in the novelty and stimulatory effect of the drug. As psychopathic individuals are characterized as novelty and sensation seekers (Cleckley, 1941; Haapasalo, 1990; Hare, 2003), the decrease in the novelty and stimulatory effect of the drug may render psychopathic individuals disinterested in the drug with an increasingly severe substance use history. Alternately, the psychopathy may suffer a deficit in cue-processing. Psychopathic individuals have commonly been noted to exhibit deficits in external stimulus and cue processing, such as through gambling tasks (Mitchell et al., 2002) or through choice-paradigms, in which the psychopath must make a decision in response to cues of both reward and punishment (Blair et al., 2006). As a result, presenting drug- and non-drug rewarding-cues in an fMRI paradigm may lack the necessary saliency for the psychopath to elicit strong neural activity that we hypothesize would be an explanatory factor for their high substance use disorder prevalence.

### Implications for Treatment

Non-invasive neurostimulation techniques have been associated with moderate success in reducing drug craving sensations. Most work to date has targeted the dorsolateral PFC in particular (Hayashi et al., 2013; Jansen et al., 2013; Shahbabaie et al., 2014), to try to increase inhibitory processing. Less work has to date targeted subcortical structures directly associated with reward processing. It may be that the regions identified as exhibiting abnormalities within our study could serve as useful targets as a means of treatment in neurostimulation protocols in individuals with substance use disorders. Considering our results, it is possible that such treatment may be successful in individuals with substance use disorders, potentially both psychopathic and non-psychopathic. This corticolimbic circuit should be investigated in terms of its implications in treatment amenability utilizing a variety of treatment strategies.

In addition, this study suggests that an externalizing behavior often associated with psychopathy could be due to neural processing biases. While it strays somewhat beyond our current data, it would also be interesting to consider whether neurostimulation protocols targeting similar regions could also benefit individuals with high levels of psychopathic traits.

### Limitations

One limitation with the current study is that we were unable to correlate psychometric craving responses with psychopathic traits or neural reactivity to food and drug stimuli due to lack of variance in craving responses. As a result, the relationship between psychopathic traits and cue-induced craving, as well as the neural underpinnings of craving, remain difficult to discern. In addition, as is common in forensic and addiction research, our study was only able to recruit a moderate sample size. Power to detect relevant effects may be particularly reduced for analyses that required separating our participants into those with high and low psychopathic traits. The modest statistical power may preclude the ability to identify smaller effect sizes. In addition, very few of our participants would be diagnosed officially as psychopathic, as it is typically required to achieve a PCL-R score of 30 to be considered psychopathic (Hare, 2003), and the highest PCL-R score within our sample was 34. However, there is a large body of research demonstrating that psychopathy is a dimensional disorder that can be conceptualized as a spectrum rather than through a categorical and dichotomized personality disorder (Neumann and Hare, 2008).

## Author Contributions

WD was responsible for the primary hypothesis of the study, the majority of the writing and data analyses, and the interpretation of these analyses for the conclusions of the study. IS contributed to the writing, table and figure composition, data analysis, and writing and revisions. MS was the laboratory director, principle investigator, and senior author of the study. MS acquired funding and acquired the majority of the data while holding a position at The Mind Research Network, and aided with writing and revisions of the study.

## Conflict of Interest Statement

The authors declare that the research was conducted in the absence of any commercial or financial relationships that could be construed as a potential conflict of interest.

## Footnotes

1 http://www.fil.ion.ucl.ac.uk/spm
