# Supplementary material for: Neuroimaging Metrics of Drug and Food Processing in Cocaine-Dependence, as a Function of Psychopathic Traits and Substance Use Severity
Source: Front Hum Neurosci. 2018 Sep 4;12:350. doi: 10.3389/fnhum.2018.00350 (PMC6132024; doi:10.3389/fnhum.2018.00350)
Supplement: Supplementary file 1 [file Data_Sheet_1.docx]

**Supplementary materials**

**Influence of PCL-R Scores and drug use severity: Non-dependent group**

The Non-dependent group, on the other hand, demonstrated no significant relationships between PCL-R scores, major drug use, and DRUG > FOOD reactivity. However, we noted significant PCL-R * Major Drug Use interaction effects in the bilateral DMPFC, right ventral striatum, and the left caudate (see Table S2). Analysis of parameter estimates demonstrated that among the High PCL-R group (PCL-R > 15), Major Drug Use positively correlated with DRUG > FOOD activity within the left DMPFC, *r* =.44, *p* = .012, right insula, *r* =.49, *p* = .005, and left caudate, *r* =.34, *p* = .058. Among the Low PCL-R group, Major Drug Use negatively correlated with DRUG > FOOD activity within the left DMPFC, *r* = -.59, *p* = .001, left ACC, *r* =-.67 *p* <.001, right ventral striatum, *r* =-.47, *p* = .014, right insula, *r* =-.34, *p* = .080, and left caudate, *r* =-.47, *p* = .014.

When correlating Major Drug Use to DRUG- and FOOD-related activity relative to baseline, the high PCL-R group demonstrated positive correlations between Major Drug Use and activity to DRUG videos within the right insula, *r* =.30, *p* = .098, and negative correlations with activity to FOOD videos within the left DMPFC, *r* =-.33, *p* = .071, and left caudate, *r* = -.43, *p* = .016. The low PCL-R group exhibited negative correlations to DRUG videos within the right insula, *r* =-.42, *p* = .029, left DMPFC, *r* =-.53, *p* = .005, right ventral striatum, *r* =-.50, *p* = .008, and left caudate, *r* =-.48, *p* = .011. In addition, they exhibit positive correlations to FOOD videos within the left DMPFC, *r* =.40, *p* = .037.

**Non-dependent Factor correlations.**

The Non-dependent group demonstrated no significant relationship between Factor 1 nor Factor 2 scores and DRUG > FOOD reactivity, however they did exhibit a significant Factor * Major Drug Use and Factor 2 * Major Drug Use interaction effects in the left DMPFC and left caudate, respectively. Among participants high in Factor 1 (Factor 1 > 5), Major Drug Use predicted an increased DRUG > FOOD reactivity within the left DMPFC, *r* = .46, *p* = .009, due to a positive correlation between Major Drug Use and DRUG-related activity relative to baseline, *r* = .40, *p* = .027. No significant correlations were observed in participants low in Factor 1 scores (Factor 1 < 5). Participants high in Factor 2 (Factor 2 > 10) exhibited a positive correlation between Major Drug Use and DRUG > FOOD reactivity within the left caudate, *r* = .51, *p* = .004, due to a negative correlation between Major Drug Use and FOOD reactivity, *r* = -.42, *p* = .022. Among participants low in Factor 2, in contrast, Major Drug Use was negatively correlated with DRUG > FOOD reactivity within the left caudate, *r* = -.51, *p* = .006, due to negative correlations to DRUG-related activity, *r* = -.54, *p* = .003.

**Appendix SA**

**Supplementary Tables**

Table S1

*Within group drug and food neural activity*

| Region | Hemi. | MNI (x, y, z) | *t* | Cluster size |
| --- | --- | --- | --- | --- |
| Food > Drug – Dependent Group | | | | |
| No significant results. |  |  |  |  |
|  |  |  |  |  |
|  |  |  |  |  |
| Food > Drug – Non-dependent Group | | | | |
| Superior parietal lobe | R | 24, -54, 63 | 5.87* | 851 |
|  | L | -18, -51, 60 | 5.58* |  |
| Posterior cingulate cortex | R | 3, -36, 39 | 3.68 |  |
| Superior occipital cortex | L | -24, -78, 30 | 5.16* | 278 |
|  | L | -36, -84, 24 | 3.90 |  |
| Superior occipital cortex | R | 18, -81, 33 | 5.05* | 542 |
| Calcarine cortex | R | 18, -48, 3 | 4.69* |  |
| Superior occipital cortex | R | 24, -81, 39 | 4.56* |  |
| Fusiform gyrus | L | -30, -42, -12 | 4.62* | 239 |
| Inferior temporal cortex | L | -63, -21, -15 | 3.93 | 165 |
|  | L | -57, -6, -24 | 3.46 |  |
| Middle temporal cortex | L | -57, -9, -9 | 3.73 |  |

| Insula | R | 48. -6, -21 | 2.84**†** | 62 |
| --- | --- | --- | --- | --- |
| Ventral striatum | R | 6, 12, -6 | 2.89**†** | 9 |
| Caudate | L | -6, -6, 9 | 2.61**†** | 25 |
|  | L | -9, -9, 12 | 2.46**†** |  |

All regions show significant activity at *p*(uncorr) < .001; * *p*(FWE) < .05; **†** *p*(svc-FWE) <.05.

Table S2

*Correlations between Total PCL-R scores and DRUG > FOOD-related hemodynamic activity among the Non-dependent group*

| Region | Hemi. | MNI (x, y, z) | *t* | Cluster size |
| --- | --- | --- | --- | --- |
| Positive – PCL-R * Major drug use | | | | |
| Caudate | L | -6, 0, 6 | 3.94 | 406 |
| Precentral gyrus | L | -15, -3, 69 | 4.84 | 559 |
|  | L | -42, 0, 57 | 4.02 |  |
| DMPFC | L | -15, 39, 45 | 4.61 |  |
| DMPFC | R | 15, 51, 21 | 4.81 | 1159 |
| Insula | R | 42, -15, -15 | 3.18† | 132 |
|  | R | 42, 0, -21 | 2.99**†** |  |
| DMPFC | L | -9, 45, 42 | 3.99**†** | 171 |
|  | L | -12, 51, 30 | 3.86**†** |  |
|  | R | 3, 48, 36 | 3.04**†** |  |
| Ventral striatum | R | 6, 12, -6 | 3.44**†** | 26 |
|  | R | 9, 9, -3 | 3.43**†** |  |
| Positive – Factor 1*Major drug use | | | | |
| DMPFC | L | -9, 42, 42 | 2.90**†** | 132 |
|  | L | -12, 48, 33 | 2.71**†** |  |
| Positive – Factor 2*Major drug use | | | | |
| Caudate nucleus | L | -9, -3, 6 | 2.98**†** | 33 |
|  | L | -6, -6, 9 | 2.88**†** |  |
|  | L | -6, 0, 9 | 2.90**†** |  |

All regions show significant activity at *p*(uncorr) < .001; * *p*(FWE) < .05; **†** *p*(svc-FWE) <.05; DMPFC = Dorsomedial prefrontal cortex.
